# Postmortem Analysis of Vitreous Urea Nitrogen, Creatinine, and Magnesium of Renal and Post-Renal Disease in Cats

**DOI:** 10.3390/toxics11080685

**Published:** 2023-08-10

**Authors:** Adam W. Stern, Daliana Roig, Courtney Valerio, Thomas Denagamage

**Affiliations:** College of Veterinary Medicine, University of Florida, Gainesville, FL 32608, USA; daliana.roig@ufl.edu (D.R.); cvalerio@ufl.edu (C.V.); tdenagamage@ufl.edu (T.D.)

**Keywords:** vitreous, postmortem, cat, creatinine, urea nitrogen, magnesium, biochemistry

## Abstract

Analysis of vitreous urea nitrogen (VUN) and creatinine (CREA) has been shown to be a useful indicator of azotemia in human postmortem examinations. Previous studies in cats, dogs, horses, and cattle have shown a good correlation between serum and postmortem ocular fluid for urea and creatinine. The aim of this study was to evaluate differences in VUN, CREA, and magnesium (MG) concentrations in vitreous humor collected postmortem from cats that presented with renal or post-renal disease and cats without renal or post-renal disease. Nine cats with renal or post-renal disease and twenty cats without renal or post-renal disease that underwent autopsy were used in this study. Collection of postmortem vitreous humor was performed, and vitreous samples were frozen at −80 °C for a minimum of 24 h prior to analysis using an in-clinic dry chemistry analyzer. Overall, there were statistically significant differences for VUN, CREA, and MG between cats with renal or post-renal disease and cats without renal or post-renal disease. Analysis of vitreous humor from cats suspected to have renal or post-renal disease can provide useful diagnostic information pertaining to renal function and issues in the urethra and urinary bladder. Future studies exploring postmortem vitreous chemistry in cats are warranted.

## 1. Introduction

Azotemia is defined as an increase in urea nitrogen and/or creatinine and can be due to pre-renal, renal, or post-renal causes. Potential causes of azotemia in cats include hypovolemia or dehydration (pre-renal azotemia), acute or chronic renal failure (renal), or post-renal obstruction (post-renal azotemia). In cats, one of the most common causes of acute renal failure is the ingestion of ethylene glycol, which is found in antifreeze and is of forensic importance given the high mortality rate in cats and ease of access to this substance.

In the clinical setting, routine biochemical analysis of antemortem serum or plasma will allow for the identification of elevations of blood urea nitrogen (BUN) and/or creatinine (CREA). Unfortunately, there are instances in which routine biochemical analysis could not be performed prior to the death of a cat including financial constraints and the sudden death of the cat. It is not possible to assess for azotemia using postmortem biochemical analysis of serum or plasma and there are no studies in cats evaluating the usage of postmortem vitreous chemistry for the diagnosis of azotemia postmortem. Comparatively in humans, postmortem vitreous humor analysis has been shown to assist pathologists in determining the cause of death [1]. For example, postmortem analysis of vitreous humor has been used for the analysis of electrolytes, creatinine, urea nitrogen, and glucose [2,3]. In cats, there is a strong correlation between serum and postmortem ocular fluid for the concentration of urea nitrogen and creatinine during the early postmortem period (at least 24 h after death) [4].

In cats, a study using the histomorphology of feline chronic kidney disease identified correlations between antemortem serum markers of renal dysfunction and identified that interstitial fibrosis best correlated with the severity of azotemia [5]. In contrast to chronic renal failure, in instances of acute renal failure, interstitial fibrosis is not a typical pathologic finding and no assumptions pertaining to azotemia from histologic examination can be made. In addition to azotemia, hypermagnesemia has been reported in cases of acute renal disease and obstructive uropathy in cats [6].

The aim of this retrospective study was to assess cats with reported vitreous urea nitrogen (VUN), CREA, and magnesium (MG) levels of vitreous humor of cats with renal or post-renal disease and compare them to cats without renal or post-renal disease (normal).

## 2. Materials and Methods

Vitreous humor was collected from cats that were presented for autopsy to the University of Florida’s Veterinary Forensic Pathology and Diagnostic Pathology services. A review of the submitted medical history and a postmortem examination was performed on each animal according to standard operating procedure to determine the cause of death. This study did not require approval from the Institutional Animal Care and Use Committee as all samples were collected postmortem and none of the animals were euthanized for purposes related to this study.

Vitreous humor was collected from each cat within 48 h of death. Vitreous humor was collected from the vitreous body following insertion of a 16-gauge needle through the lateral aspect of the sclera and gentle aspiration into a 3 mL syringe. Samples were put into a sterile serum activator tube (Becton Dickinson, East Rutherford, NJ, USA). Samples were frozen at −80 °C for a minimum of 1 h prior to analysis. Only samples free of blood contamination were used. Samples were prepared through thawing and then spun in a microcentrifuge (Heska Corporation, Loveland, CO, USA) for 5 min, and the supernatant was drawn up and placed in a 0.5 mL red-top tube (Heska Corporation, Loveland, CO, USA) for analysis. Fluid analyses included VUN (mg/dL), CREA (mg/dL), and MG (mg/dL). Analytes were measured on a Heska Element DC Veterinary Chemistry Analyzer (Heska Corporation, Loveland, CO, USA) according to the manufacturer’s instructions for serum samples. If test results exceeded the measuring range (defined by the manufacturer), the vitreous sample was diluted according to the manufacturer’s instructions using a dilution factor (1:4) and re-measured. The sample dilution could not be carried out for one case as the entire sample was consumed during initial testing and the test result exceeding the measuring range was assigned the maximum quantitative value. The magnesium concentration for one cat was not measured.

Data normality was assessed for VUN, CREA and MG in diseased and normal cats by using the Shapiro–Wilk normality test. Descriptive statistics were calculated to present the mean and standard deviation. Two-sample *t*-test was used to compare mean differences of VUN, CREA, and MG levels between cats with renal or post-renal disease and normal cats. Results are presented as mean difference, standard error of the mean difference, and *p*-value. Tests were considered significant if *p* < 0.05. Statistical analysis was performed using Statistix 10 (Analytical Software, Tallahassee, FL, USA).

## 3. Results

Twenty-nine adult cats were used in this study. Nine cats had renal or post-renal disease including renal papillary necrosis, acute renal failure due to ethylene glycol intoxication, or obstructive uropathy. Twenty cats that did not have renal or post-renal disease and died from non-renal causes including vehicular trauma or predation were used for comparison.

VUN and CREA were higher in the cats with renal or post-renal disease than in the cats without renal or post-renal disease. The MG levels in four out of eight cats with renal or post-renal disease were higher than those in the cats without renal or post-renal disease. The mean VUN, CREA, and MG in the cats with renal or post-renal disease were 245.56 mg/dL, 13.92 mg/dL, and 3.46 mg/dL, respectively (Table 1). The mean VUN, CREA, and MG in the cats without renal or post-renal disease were 23.65 mg/dL, 1.03 mg/dL, and 1.61 mg/dL, respectively (Table 1). Results of the two-sample *t*-test were statistically significant for the differences between the VUN (*p* = 0.0003), CREA (*p* = 0.0002), and MG (*p* = 0.04) in the cats with renal or post-renal disease compared to cats without renal or post-renal disease. A preliminary sample size calculation was not performed. However, post hoc power analysis for two-sample *t*-tests showed 99% and 100% power to detect VUN and CREA, respectively, in the samples selected in this study, while the power to detect MG was lower at 57%.

## 4. Discussion

This study evaluated the differences between VUN, CREA, and MG measurements collected from the vitreous body in cats with renal and post-renal disease and compared the results to cats without renal and post-renal disease. A comparison of VUN and CREA from samples collected from cats with renal or post-renal disease revealed higher levels of both analytes could be detected in the vitreous humor, similar to the results of antemortem biochemical analysis of serum or plasma. Half of the cats with renal or post-renal disease had elevated levels of MG within the vitreous humor. The results of this study suggest that in deceased cats with renal or post-renal disease, VUN and CREA are likely to be elevated, and concurrently, MG could also be elevated. The ability to perform biochemical analysis of vitreous humor using samples collected at autopsy will be useful to assess renal function rather than relying solely on the subjective assessments of histopathology of the kidney.

Postmortem vitreous chemistry in human medicine is considered a valuable tool to help support diagnoses made at autopsy as different analytes can be helpful in determining cause of death [2,3]. Multiple studies have concluded that urea nitrogen is stable and “closely approximate the antemortem serum levels” [2]. It has been shown that VUN, CREA, and uric acid can all be used to diagnose chronic kidney disease and end-stage renal failure in humans and are considered relatively stable analytes postmortem [7]. A comparison of antemortem serum and the postmortem vitreous humor in cats, dogs, and cattle showed a strong correlation between serum and postmortem eye fluid for urea nitrogen and CREA collected at necropsy at least 24 h after death [4]. In cattle, it has been shown that vitreous humor can be used to detect magnesium imbalances for at least 48 h postmortem [8] The postmortem analysis of vitreous humor from eight cows with uremia showed that postmortem analysis of vitreous humor could be successfully used to diagnose azotemia [9]. In the current study, 100% of cats with elevated VUN and CREA died from renal or post-renal disease.

Magnesium is considered to be the “forgotten” ion. A postmortem study in humans showed that vitreous humor MG did not correlate with age, postmortem interval, or other vitreous electrolytes and was stable during the early postmortem period (up to 75 h postmortem) [10]. In a feline study, serum hypermagnesemia was observed in 16/42 (38.1%) and hypomagnesemia in 6/42 (14.3%) cats with chronic renal disease [11]. In another study, hypermagnesemia was detected only in cats with renal disease, obstructive uropathy, or neoplastic disease [6]. In the current study, 50% of cats developed hypermagnesemia within the vitreous humor, and these cats had renal papillary necrosis (one cat), ethylene glycol toxicity (two cats), and urethral obstruction (one cat). It is possible that diffusion of MG into the vitreous humor takes time, and since the time of onset for disease was not known for all cases, it is possible that if the cats had survived longer, vitreous MG levels would have become elevated.

The Heska Element DC Veterinary Chemistry Analyzer has been shown to be able to analyze postmortem vitreous humor from cats [12]. Although there are no standard reference intervals for VUN, CREA, or MG in cats, we found that normal cats, those without renal or post-renal disease, had a mean VUN level of 23.47 mg/dL (range: 14.6–32.6 mg/dL), a mean CREA level of 1.61 mg/dL (range: 0.2–2.3 mg/dL), and a mean MG level of 1.26 mg/dL (range: 0.3–2.3 mg/dL). The range of VUN in these normal cats was similar to the reported reference ranges for BUN (15–32 mg/dL) according to the Heska Element DC Veterinary Chemistry Analyzer. The range of CREA and MG in normal cats was at or below the reported reference range for CREA (0.8–1.8 mg/dL) and MG (1.8–2.7 mg/dL) according to the Heska Element DC Veterinary Chemistry Analyzer. Although the normal ranges of CREA and MG tend to overlap lower levels, this does not affect the possibility of utilizing these analytes to assess for azotemia or hypermagnesemia.

## 5. Conclusions

This study indicated that cats with renal or post-renal disease, such as ethylene glycol toxicity or urethral obstruction, can have significant VUN, CREA, and occasionally MG elevations compared to cats that do not have renal or post-renal disease. The results of this study provide evidence that the pathologist can use feline vitreous humor to assess for antemortem azotemia using vitreous chemistry rather than hypothesize that changes within the kidneys could have resulted in azotemia. Although we were able to detect a statistically significant difference between VUN, CREA, and MG measurements in these cats, a larger sample size could allow further statistical analyses and the grouping of patients by various disease processes. Therefore, future larger studies analyzing postmortem vitreous humor in cats as well as other species, including dogs, cattle, horses, and marine mammals, are needed. A recent study using delphinid vitreous humor demonstrated that the Abaxis VetScan VS2 (VetScan) chemistry analyzer had acceptable precision for multiple analytes [13], and marine mammals should be considered. Another area of future study should include the effects of storage (freezing) and decomposition of animals as it is not uncommon for bodies to be frozen for storage or for animals to be discovered several days after their death. Additionally, the assessment of cats with clinically documented pre-renal azotemia such as dehydration would also be beneficial as dehydration is a difficult diagnosis to make solely based on autopsy findings. Another process to explore is fatal cases of heatstroke in cats and dogs since in humans, Zhu et al. showed that elevated vitreous CREA levels can reflect skeletal muscle damage in cases of heat stroke [14]. Further vitreous chemistry research would benefit veterinary pathologists performing autopsies as vitreous chemistry can be utilized to assess for clinically relevant azotemia.

## Figures and Tables

**Table 1:** Descriptive statistics of VUN, creatinine, and magnesium levels in renal/post-renal disease and control cats, and mean difference, SE, and *p*-value for group differences.

	Disease Cats			Control Cats					
Variable	N	Mean	SD	N	Mean	SD	Mean Difference	SE	*p*-Value *
VUN	9	245.56	112.14	20	23.65	5.93	221.9	24.58	0.0003
Creatinine	9	13.92	6.14	20	1.03	0.67	12.8	1.36	0.0002
Magnesium	8	3.46	2.13	20	1.61	0.51	1.8	0.49	0.04

VUN = vitreous urea nitrogen, SD = standard deviation, SE = standard error; * *p*-value for two-sample *t*-test is <0.05.

## Data Availability

The data that support the findings of this study are available from the corresponding author, A.W.S., upon reasonable request.
